# Cigarette smoke condensate increases *C. albicans* adhesion, growth, biofilm formation, and *EAP1*, *HWP1* and *SAP2* gene expression

**DOI:** 10.1186/1471-2180-14-61

**Published:** 2014-03-12

**Authors:** Abdelhabib Semlali, Kerstin Killer, Humidah Alanazi, Witold Chmielewski, Mahmoud Rouabhia

**Affiliations:** 1 Groupe de Recherche en Écologie Buccale, Faculté de Médecine Dentaire, Université Laval, 2420 rue de la Terrasse, Québec G1V 0A6, Canada; 2 Genome Research Chair, Department of Biochemistry, College of Science King Saud University, Riyadh, Kingdom of Saudi Arabia

**Keywords:** Cigarette smoke, Tobacco, *C albicans*, Adhesion, Growth, Biofilm, Genes, *EAP-1*, *HWP-1*, *Sap2*

## Abstract

**Background:**

Smokers are more prone to oral infections than are non-smokers. Cigarette smoke reaches the host cells but also microorganisms present in the oral cavity. The contact between cigarette smoke and oral bacteria promotes such oral diseases as periodontitis. Cigarette smoke can also modulate *C. albicans* activities that promote oral candidiasis. The goal of this study was to investigate the effect of cigarette smoke condensate on *C. albicans* adhesion, growth, and biofilm formation as well as the activation of *EAP1*, *HWP1* and secreted aspartic protease 2.

**Results:**

Cigarette smoke condensate (CSC) increased *C. albicans* adhesion and growth, as well as biofilm formation. These features may be supported by the activation of certain important genes. Using quantitative RT-PCR, we demonstrated that CSC-exposed *C. albicans* expressed high levels of *EAP1*, *HWP1* and *SAP2* mRNA and that this gene expression increased with increasing concentrations of CSC.

**Conclusion:**

CSC induction of *C. albicans* adhesion, growth, and biofilm formation may explain the increased persistence of this pathogen in smokers. These findings may also be relevant to other biofilm-induced oral diseases.

## Background

One of the most commonly encountered opportunistic microorganisms in humans is *Candida albicans,* a ubiquitous fungus that is a part of the normal microbial flora found on mucosal surfaces such as those of the oral cavity, gastrointestinal tract, and vagina in human beings and domestic animals [1]. This yeast is the most common cause of mucosal and invasive fungal infections observed in humans [2]. Host protection against *C. albicans* infection is complex and includes different subsets of the immune defense system [3-5].

During the development of oropharyngeal candidiasis (OPC), *Candida* adheres to and invades the tissue. The adhesion of this yeast to host tissue is the initial phase of a potential infection that enables microorganisms to survive inside the host and eventually colonize host tissues during the onset of candidiasis [6,7]. Following this irreversible attachment, fungal cells proliferate and optimally interact to ensure their sustainability in the imposed environment. After the proliferative phase comes the production and deposition of a thick extracellular matrix (mature biofilm) that procures chemical as well as physical protection for cells [8,9].

*C. albicans* adhesion and growth leading to biofilm formation are mediated by various genes such as *HWP1* and *EAP1* that encode well-characterized class of cell wall proteins that house a glycosylphosphatidylinositol (GPI) anchorage motif in their C–terminal domains and a signal peptide at their N-termini. These C- and N-terminal domains mediate the adherence of *C. albicans* to various surfaces [10]. *HWP1,* a hyphal-specific adhesion gene encodes the hyphal cell wall protein, and both are essential for biofilm formation [11]. The involvement of *HWP1* in *C. albicans* adhesion can be supported by the *EAP1* gene which encodes a glucan-cross-linked cell wall protein (adhesin Eap1p) and mediates the adhesion of *C. albicans* to different surfaces, including epithelial cells and polystyrene [12]. Similar to other genes, *HWP1* and *EAP1* are downstream effectors of *EFG1*[13], a transcription regulator [14]. The *efg1 mutant* strain has been shown to exhibit defects in growth, biofilm formation, and virulence [15].

*C. albicans* virulence is also mediated by proteolytic enzymes such as secreted aspartyl proteinases (Saps) [16,17]. The contribution of Saps to mucosal and systemic infections and their involvement in adherence, tissue damage, and evasion of host immune responses has been reported, showing the implication of the Sap2 gene in *C. albicans* growth in protein-containing media [18]. SAP1 and SAP3 are expressed during phenotypic switching [19,20], while SAP4, SAP5 and SAP6 are expressed upon hyphal formation [20]. SAP9 and SAP10 are involved in the mechanism of adhesion to host cells [21]. This proteolytic enzyme family is therefore involved in *C. albicans* virulence.

*C. albicans* infection can be promoted by several factors. Candidiasis has been associated with long-term antibiotics intake, AIDS, leukemia, malignancy, radiation therapy for head and neck cancer, or other risk factors that interfere with immunocompetence [22-24].

Environmental factors such as smoking may also promote *Candida* infections [25,26]. Tobacco smoke exposure has been shown to promote microbial biofilm formation [27]. Specifically, it has been shown that cigarette smoke interferes with *S mutans* and *C. albicans* adhesion, resulting in biofilm formation on dental restoration materials [28], which suggests that cigarette smokers are more susceptible to life-threatening oral infections including candidiasis. The aim of this study was to investigate the effect of cigarette smoke condensate on *C. albicans* adhesion, growth, and biofilm formation, and on the activation of several genes involved in the virulence of this yeast.

## Results

### Cigarette smoke condensate promoted *C albicans* adhesion and growth

**Figure 1:**
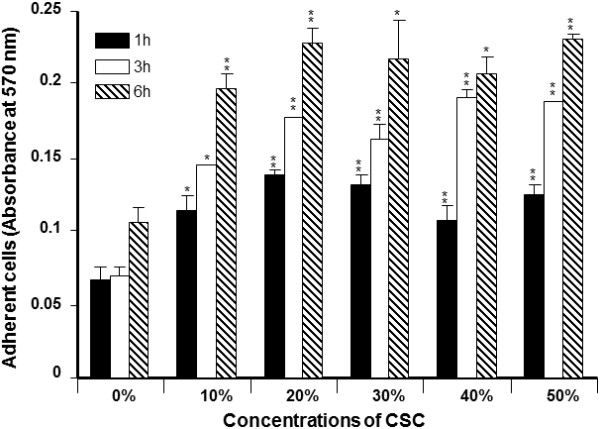
**Effect of CSC on *C. albicans *adhesion.** Yeast cells were grown on glass slides with or without various concentrations of CSC for different time points at 30°C. The attached cells were stained with crystal violet and read with an ELISA reader at 570 nm (n = 5). *p < 0.05; **p < 0.01, compared to the non-CSC-cultured *C. albicans*.

**Figure 2:**
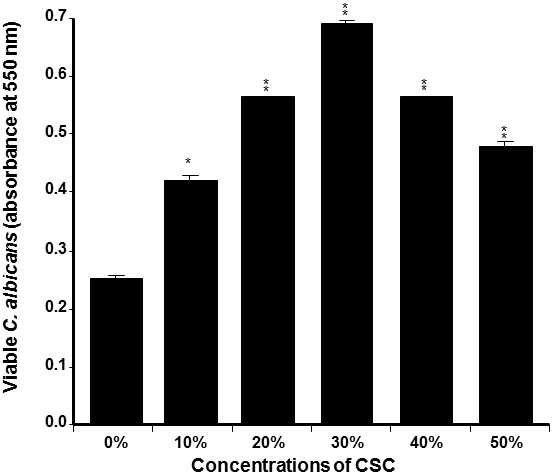
**CSC promoted *C. albicans *growth.** Yeast cells were seeded with or without CSC at various concentrations. The cultures were maintained for 24 h at 30°C. The live cells were ascertained by MTT assay (n = 4). *p < 0.05; **p < 0.01, compared to the non-CSC-cultured *C. albicans.*

*C. albicans* attachment to the surface of glass slides for 1, 3, and 6 h was measured by means of crystal violet staining. As shown in Figure 1*C. albicans* adhesion was significant (p < 0.05) at 3 and 6 h of incubation. All of the tested CSC concentrations promoted the adhesion of *C. albicans*. Adhesion was related to incubation period, with low adhesion reported at 1 h and high adhesion at 6 h. This result indicates that CSC can increase *C. albicans* adhesion and that this effect can lead to significant *C. albicans* growth. For this purpose, we investigated the effect of CSC on *C. albicans* growth. As shown in Figure 2, high *C. albicans* growth was obtained in the presence of CSC, compared to that obtained by the controls. Indeed, *C. albicans* growth significantly (P < 0.05) increased with as low as 10% CSC. Of interest is that the most effective concentrations of CSC were between 20 and 40%; at these concentrations, *C. albicans* growth was two to threefold higher than that recorded by the controls. Overall data thus demonstrate that cigarette smoke favors *C. albicans* adhesion and growth.

### Cigarette smoke condensate promoted *C albicans* biofilm formation

**Figure 3:**
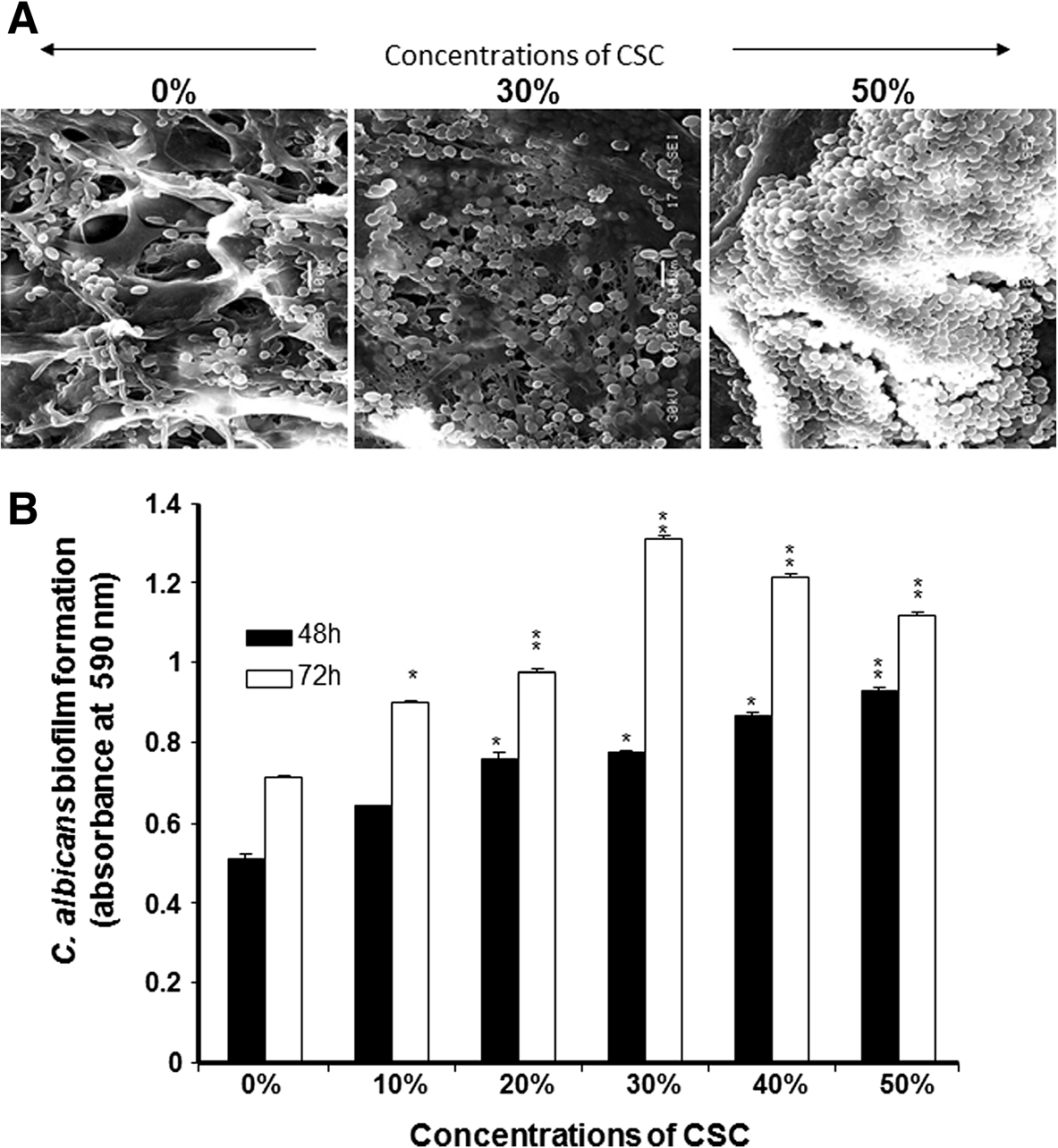
**CSC promoted *C. albicans *biofilm formation.** Yeast cells were grown with or without CSC at various concentrations for 48 and 72 h at 30°C. Panel **A**: Biofilms examined by SEM; photos are presented (n = 4). Panel **B**: *C. albicans-*seeded membranes stained with crystal violet. The optical density of each solution was read at 570 nm by means of a plate reader (n = 6). *p < 0.05; **p < 0.01, compared to the non-CSC-cultured *C. albicans.*

Because CSC contributed to increasing *C. albicans* adhesion and growth, we tested its potential to promote *C. albicans* biofilm formation. Using SEM analyses and a crystal violet assay, we were able to demonstrate the stimulatory effect of CSC on biofilm formation (Figure 3). SEM analyses revealed a high *C. albicans* density in the CSC-treated culture (Figure 3A). A high *C. albicans* density was observed in the scaffold in the presence of 30% CSC and this density increased with 50% CSC. To confirm these observations, quantitative analyses were conducted using the crystal violet staining method. Figure 3B showed that after 2 days of culture, CSC was able to significantly (p < 0.05) increase biofilm formation. This effect was observed beginning at a concentration of 20% CSC, and at 50% CSC, biofilm formation was greater than that observed in the controls and at 30% CSC.

### Cigarette smoke condensate modulated *HWP1*, *EAP1*, and *SAP2* expression

**Figure 4:**
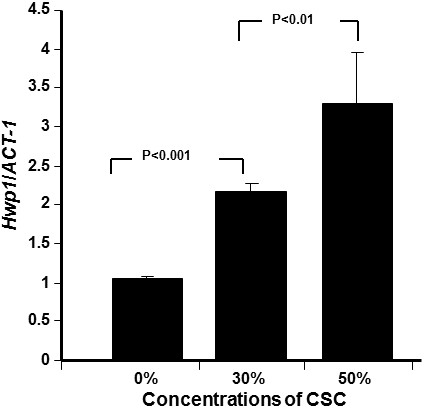
***HWP1 *mRNA expression by *C. albicans *increased with CSC stimulation.** Yeast cells were grown with or without CSC (30 and 50%) for 24 h. *HWP1* gene expression was analyzed by qRT-PCR (n = 5).

**Figure 5:**
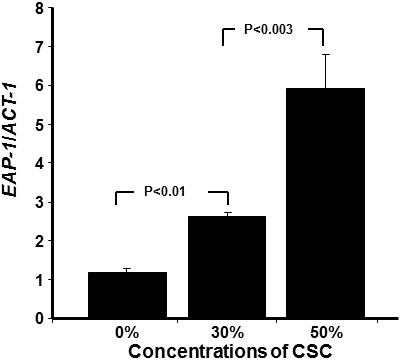
**Cigarette smoke condensate induced *EAP1 *mRNA expression by *C. albicans*.** Yeast cells were grown with or without CSC (30 and 50%) for 24 h. *EAP1* gene expression was analyzed by qRT-PCR (n = 6).

**Figure 6:**
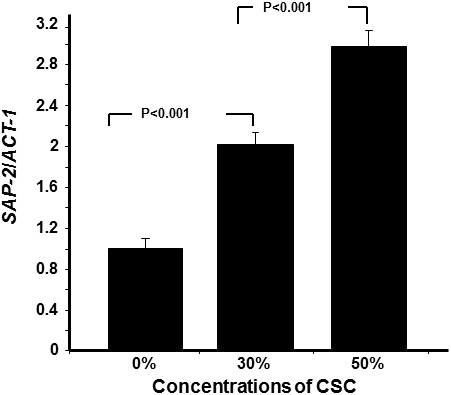
***C. albicans *expressed high levels of *Sap2 *mRNA following exposure to CSC.** Yeast cells were grown with or without CSC (30 or 50%) for 24 h prior to RNA extraction and *Sap2* mRNA expression analysis by qRT-PCR (n = 5).

Based on the data showing that CSC increased *C. albicans* adhesion, growth, and biofilm formation, we sought to determine whether this took place through the regulation of certain genes. Figure 4 reveals that *HWP1* gene expression significantly increased following exposure of *C. albicans* to CSC. The activation of this gene significantly (p < 0.001) increased according to CSC concentration. As shown in Figure 4, a twofold increase in *HWP1* gene expression was recorded with a concentration of 30% CSC, compared to that observed in the controls, and with 50% CSC, this increase was over threefold. Similarly, *EAP1* gene, which encodes a glycosylphosphatidylinositol-anchored, glucan-cross-linked cell wall protein involved in adhesion and biofilm formation, was also affected by CSC treatment. Figure 5 shows that CSC significantly increased the expression of the *EAP1* gene and that this increase was dependent on the concentration of CSC; the higher the concentration, the greater was the expression of this gene. The *SAP2* gene was also modulated by CSC. Figure 6 shows that the CSC led to a significant (p < 0.001) increase of *SAP2* gene expression. Of interest is the increased *Sap2* gene expression with CSC concentration. Indeed, with 30% CSC, a twofold expression was recorded and with 50% CSC this expression increased threefold compared to that observed in the controls (*C. albicans* not exposed to CSC).

## Discussion

Smoking is known to induce a variety of changes in the oral cavity. Cigarette smoke affects both saliva [29] and oral microorganisms, including *C. albicans,* a leading cause of oral candidiasis [28]. However, the specific effect of cigarette smoke on *C.albicans* remains to be elucidated. The first question we addressed in this study was: What is the effect of cigarette smoke on *C. albicans* adhesion? We demonstrated that CSC promoted *C. albicans* adhesion, which is in agreement with previously reported studies showing a high rate of oral candida carriage in tobacco smokers compared to non-smokers [30,31]. Furthermore, bacteria exposed to CSC were shown to adhere more to epithelial cells compared to non-exposed specimens [32], which supports our data with *C. albicans*. Increased adhesion of *C. albicans* in the presence of CSC may occur due to changes in the interaction of *C. albicans* with its environment through the expression of high levels of adhesins, as previously suggested [33]. Furthermore, it is important to realize that cigarette smoke comprises a high number of individual compounds [34], including acetaldehyde, benzene, 1,3-butadiene, and isoprene with an elevated mutagenic potential [35]. Thus, it is possible that these compounds may have exerted specific effect on *C. albicans* adhesion, growth and probably biofilm formation. The effect of cigarette smoke promoting cell adhesion was previously reported by Baboni et al., (2009) showing linear dose response adhesion [25]. The mechanism involved in such effect could involve kinase pathways. These pathways can be promoted by CSC compounds at certain concentration, but inhibited when these compounds are high explaining the decrease of *C. albicans* adhesion/biofilm formation at 40% and 50% of CSC. Further research is mandatory to shed light on the mechanisms leading to the up-regulation of *C. albicans* adhesion when exposed to cigarette smoke.

*C. albicans* adhesion is one of the key events leading to candidiasis [36,37]. This adhesion is usually followed by overgrowth and invasion [38]. Consequently, in promoting *C. albicans* adhesion, CSC may lead to an over-growth of this yeast. Our study confirms this hypothesis showing a growth increase of *C. albicans* in the presence of CSC. This concurs with previously published reports showing that smoking can be an important predisposing factor for oral candidiasis [28], which may be enhanced by cigarette smoke through an increased secretion of histolytic enzymes by *C. albicans*, thus contributing to its virulence [28]. However, the exact pathogenic influence of smoking has yet to be investigated.

*C. albicans* adhesion and growth are particularly necessary for biofilm formation [39,40]. Because CSC significantly increased *C. albicans* adhesion and growth in the present study, it is suggested that CSC may also promote *C. albicans* biofilm formation. Using appropriate conditions to form biofilms, our findings indicate that CSC was indeed capable of promoting biofilm formation. Of interest is that a significant increase of biofilm formation was obtained at both tested concentrations, and that this phenomenon was dependent on CSC concentration. These useful data are comparable to those of other studies showing increased microbial biofilm formation with cigarette smoke [41-43]. By showing the significant stimulatory effect on increasing *C. albicans* biofilm formation, cigarette smoke can thus be labeled as an infection-promoting agent.

Promoting *C. albicans* adhesion, growth, and biofilm formation may operate through the modulated expression of certain *C. albicans* genes [44,45], as supported by our study demonstrating that CSC led to a high expression of the *EAP1* gene. As a member of the GPI-CWP family in *C. albicans*[46], Eap1p was originally identified because of its ability to mediate adhesion to polystyrene when the *EAP1* gene was expressed in a flocculin-deficient *Saccharomyces cerevisiae* strain. *EAP1* expression in a *C. albicans efg1/efg1* mutant was able to restore *C. albicans* adhesion to epithelial cells [12]. Deleting *EAP1* in *C. albicans* was shown to reduce cell adhesion to polystyrene and to epithelial cells in a gene dosage-dependent manner [12,46]. Indeed, this suggests that exposure to CSC increases *EAP1* expression, which may in turn contribute to increasing *C. albicans* adhesion, and ultimately, biofilm formation and pathogenesis.

We also demonstrated that CSC increased *HWP1* mRNA expression. *HWP1* is a downstream component of the cAMP-dependent PKA pathway and is positively regulated by *EFG1*[47]. The transcript level of *HWP1* increased with increasing CSC stimulation, which suggests that CSC did affect cAMP–*EFG1* pathway activity, resulting in an increase of *C. albicans* adhesion and growth with biofilm formation. Further investigations are therefore warranted to gain greater insight into the interaction between cigarette smoke and *C. albicans* leading to infection.

*Candida* pathogenesis is associated with the production and secretion of histolytic enzymes [48]. Secreted aspartyl proteases (Saps) and phospholipases were specifically reported as being directly related to *C. albicans* virulence [49]. During infection, Saps are incriminated degrading host proteins involved in tissue barriers and immune defense [18,50,51]. Here, we report that CSC upregulated Sap2 mRNA expression. It is known that Sap gene upregulation contributes to increasing *C. albicans* transition, and later, its pathogenicity through an augmented secretion of proteinases [52]. Our study thus establishes, for the first time, a clear link between cigarette smoke and *C. albicans* pathogenesis through the behavior of key genes such as *EAP1*, *HWP1* and *Sap2*. These genes are known to be involved in controlling *Candida* adhesion, growth, and biofilm formation [53]; however, the precise interactions between these different genes and cigarette smoke during *C. albicans* pathogenesis have not yet been fully investigated. Data suggest that gene activation can be involved in *C. albicans* adhesion, growth and biofilm formation promoted by CSC. This may involve kinase pathways contributing to *C. albicans* adaptation to the CSC environment as previously suggested [25].

## Conclusions

This study demonstrated that CSC upregulates *C. albicans* adhesion and growth that promote biofilm formation. Of interest is that these effects were supported by the modulation of *C. albicans* genes *EAP1*, *HWP1*, and *Sap2*. Overall results therefore suggest a possible link between cigarette smoke, *C. albicans* activation, and oral candidiasis.

## Methods

### Preparation of cigarette smoke condensate

**Figure 7:**
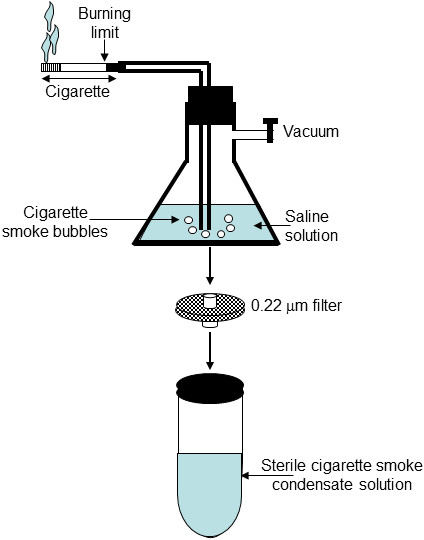
**Generation of sterile CSC.** Smoke from a burning cigarette was passed through a 0.9% saline solution. The CSC-rich solution was then sterilized by filtration through 0.22-um sterile filters and later stored at -20°C until use.

1R3F cigarettes were purchased from the Kentucky Tobacco Research & Development Center (Orlando, FL) and were used to prepare the cigarette smoke condensate solution, as shown in Figure 7. Each cigarette was placed into one end of a silicone tube linked to an Erlenmeyer flask containing 200 ml of 0.09% sodium chloride. On the other end, a second silicone tube linked to the Erlenmeyer was connected to a standard vacuum. The cigarette was attached to the cigarette holder and lit and the smoke was extracted by applying vacuum, pulling the smoke directly into the 0.09% sodium chloride solution. The process was repeated for a total of ten whole cigarettes. The resulting cigarette smoke condensate (CSC) solution was then sterilized by filtration through a 0.22 μm filter and stored at 4°C until use.

### *Candida* strain

*C. albicans* SC5314 was cultured for 24 h on Sabouraud dextrose agar plates (Becton Dickinson, Oakville, ON, Canada) at 30°C. For the *C. albicans* suspensions, one colony was used to inoculate 10 ml of Sabouraud liquid medium supplemented with 0.1% glucose, pH 5.6. The cultures were then grown to the stationary phase in a shaking water bath for 18 h at 30°C, after which time the yeast cells were collected, washed with phosphate-buffered saline (PBS), counted by means of a haemocytometer, and adjusted to 10^6^/ml prior to use.

### Effect of CSC on C albicans adhesion

A glass slide (0.5 cm in diameter) was placed into each well of a 12-well plate then covered with 1 ml of sterile artificial saliva solution at room temperature for 30 min under agitation to ensure that the slides were covered by liquid at all times. Before use, the artificial saliva (2.5 g of NaCl, 332.97 mg of CaCl_2_, 250 mg of MgCl_2_.6H_2_O, 189.48 mg of KCl, 3 g of anhydrous potassium acetate (C_2_H_3_O_2_K), 772.00 mg of K_3_PO_4_.3 H_2_O, and 0.1 ml of H_3_PO_4_ (85 wt. % in H_2_O, Sigma Aldrich)) was supplemented with 140 mg of Type II mucin in 1000 ml of the prepared saliva solution, pH 7. Following the saliva coating, the glass slides were gently rinsed with sterile saline, transferred to new wells of sterile 12-well plates, and subsequently dried for 3 h under a sterile culture hood. Each saliva-coated glass slide was then covered with 10^4^*C. albicans* cells in 50 μl of Sabouraud medium supplemented or not with CSC at various concentrations (10, 20, 30, 40 or 50%) and incubated for 60 nm at 30°C under stable conditions to prevent the medium from leaking from the slide onto the plastic. Following this incubation period, 1 ml of Sabouraud with or without CSC was added to each well and the cultures were further incubated for 1, 3, or 6 h. At the end of each incubation period, each slide was removed from its well and placed into new wells of a 12-well plate, subsequently washed twice with warm PBS, and subjected to crystal violet staining. One milliliter of 1% w/v crystal violet solution in demineralized water was added, and the slides were further incubated at room temperature for 30 min. After incubation, the non-bound dye was removed from the wells by thorough washing with demineralized water, followed by drying at 37°C. Bound crystal violet was dissolved by adding 1 ml of absolute ethanol and incubating on a rocking platform for 15 min at room temperature. The absorbance levels of the dissolved dye were measured at a wavelength of 590 nm by means of an optical density reader (X-Mark Microplate Spectrophotometer, Bio-Rad Laboratories, Mississauga, ON, Canada).

### Effect of CSC on C albicans growth

The yeast was seeded into separate tubes (10^5^*C. albicans* per tube) in 4 ml of Sabouraud culture medium supplemented or not with CSC at 10, 20, 30, 40, or 50%. The cultures were maintained at 30°C for 24 h, after which time *C. albicans* growth was evaluated by means of the (3-(4,5-dimethylthiazol-2-yl)-2,5-diphenyl tetrazolium bromide) (MTT) assay (Sigma-Aldrich, St. Louis, MO) which measures viable cells as a function of mitochondrial activity. Briefly, an MTT stock solution (5 mg/ml) was prepared in PBS and added to each culture at a final concentration of 10% (v/v). The *C. albicans* cultures were then incubated with the MTT solution at 30°C for 4 h, after which time the plate was centrifuged for 10 min at 1200 rpm and the supernatant was removed. The remaining pellet in each condition was then washed with warm PBS, and 2 ml of 0.04 N HCl in isopropanol were added to each pellet, with a further incubation for 15 min. Absorbance (optical density, OD) was subsequently measured at 550 nm by means of an xMark microplate spectrophotometer (Bio-Rad, Mississauga, ON, Canada).

#### Effect of CSC on *C albicans* biofilm formation

*C. albicans* biofilms were obtained by culturing the yeast on a porous collagen scaffold which facilitated *C. albicans* penetration through the pores and its adhesion to the scaffold through collagen affinity. This also promoted biofilm formation and handling with no cell loss, thus contributing to the maintenance of the biofilm structure. For this purpose, 5 mm × 5 mm samples of porous scaffold (Collatape, Zimmer Dental Inc., Carlsbad, CA, USA) were placed in a 24-well plate, rinsed twice with culture medium, seeded with *C. albicans* (10^6^ cells), and finally incubated for 30 min at 30°C without shaking to allow for adherence. Fresh Sabouraud medium was added to each well in the presence or absence of various concentrations of CSC (10, 30, or 50%). The *C. albicans*-seeded scaffolds were then incubated for 2 or 3 days at 30°C. Following each culture period, *C. albicans* growth and biofilm formation was assessed by scanning electron microscopy and the crystal violet assay.

### Scanning electron microscopy (SEM) analysis

*C. albicans-*rich scaffolds (biofilms) were fixed in ethylene glycol for 60 min followed by a rinse with sterile PBS. Dehydration was performed in a series of 5-min treatments with ethanol solutions of increasing concentration (50, 70, 90, and twice at 100%). The dehydrated biofilms were kept overnight in a vacuum oven at 25°C, after which time they were sputter-coated with gold, examined, and imaged under a JEOL 6360 LV SEM (Soquelec, Montréal, QC, Canada) operating at a 30 kV accelerating voltage.

### Biofilm staining by crystal violet

Following incubation for 24 h at 30°C, biofilms were stained with 1% crystal violet (100 μl) for 15 min. They were then washed three times with PBS to remove unbound crystal violet dye and were dried overnight at room temperature. The biofilms were then covered with 1 ml of absolute ethanol and were incubated on a rocking platform for 20 min at room temperature to release the stain from the biofilms. Absorbance was recorded at 590 nm. Each biofilm assay was run in triplicate and the means ± standard deviations of four separate experiments were calculated and plotted.

#### Effect of CSC on *C albicans* gene activation/repression

*C. albicans* (10^5^ cells) was cultured in the presence or absence of CSC at various concentrations (30 and 50%) at 30°C for 24 h under agitation. At the end of this incubation period, the cultures were centrifuged 10 min at 13,000 rpm, the supernatants were discarded, and each pellet was suspended in 0.6 ml of lysis buffer (1 M glycerol, 0.1 M EDTA). Glass beads (0.425-0.6 mm in diameter; 0.2 ml) were then added to each suspended pellet prior to sonication (4 × 1 min, followed by 2 min of incubation in ice) by means of a MiniBead-beater (Biospec Products, Bartlesville, OK, USA). Following cell lysis, the total RNA was extracted from each sample by means of the Illustra RNAspin Mini kit (GE Health Care UK Limited, Buckingham, UK). The concentration, purity, and quality of the extracted RNA were determined using the Experion system and the RNA StdSens analysis kit according to instructions provided by the manufacturer (Bio-Rad, Hercules, CA, USA). Appropriate RNAs were used to perform quantitative RT-PCR.

#### Quantitative real-time RT-PCR

RNA (500 ng of each sample) was reverse transcribed into cDNA by means of the iScript cDNA synthesis kit (Bio-Rad, Mississauga, ON, Canada). The conditions for the preparation of the cDNA templates for PCR analysis were 5 min at 25°C, 1 h at 42°C, and 5 min at 85°C. Quantitative PCR (qPCR) was carried out. The quantity of mRNA transcripts was measured with the Bio-Rad CFX96 real-time PCR detection system. Reactions were performed using a PCR supermix (iQ SYBR Green Supermix, Bio-Rad). Primers (Table 1) were added to the reaction mix to a final concentration of 250 nM. Five microliters of each cDNA sample was added to a 20 μl PCR mixture containing 12.5 μl of the iQ SYBR Green supermix, 0.5 μl of specific primers *ACT1, SAP2, HWP1,* and *EAP1* (Invitrogen Life Technologies Inc., Burlington, ON, Canada), and 7 μl of RNase/DNase-free water (MP Biomedicals, Solon, OH, USA). Each reaction was performed in a Bio-Rad MyCycler thermal cycler. For the qPCR, the CT was automatically determined by the accompanying Bio-Rad CFX manager. Thermocycling conditions for the *ACT1*, *Sap2*, and *EAP1* were established at 5 min at 95°C, followed by 30 cycles of 15 s at 95°C, 30 s at 60°C, and 30 s at 72°C, with each reaction performed in triplicate. For the *HWP1*, the thermocycling conditions were 3 min at 95°C, followed by 30 cycles of 10 s at 95°C, 30 s at 54°C, and 40 s at 72°C, with each reaction also performed in triplicate. The specificity of each primer pair was determined by the presence of a single melting temperature peak. The *ACT1* produced uniform expression levels varying by less than 0.5 CTs between sample conditions and thus became the reference gene for this study. The results were analyzed using the 2^-ΔΔCt^ (Livak) relative expression method.

**Table 1:** Primer sequences used for the qRT-PCR

**Gene**	**Primer sequence 5′ to 3′**	**Amp size (bp)**
***ACT1***	Forward: GCTGGTAGAGACTTGACCAACCA	87
	Reverse: GACAATTTCTCTTTCAGCACTAGTAGTGA	
***EAP1***	Forward: CTGCTCACTCAACTTCAATTGTCG	51
	Reverse: GAACACATCCACCTTCGGGA	
***HWP1***	Forward: GCTCAACTTATTGCTATCGCTTATTACA	67
	Reverse: GACCGTCTACCTGTGGGACAGT	
***SAP2***	Forward: TCCTGATGTTAATGTTGATTGTCAAG	82
	Reverse: TGGATCATATGTCCCCTTTTGTT	

### Statistical analysis

Each experiment was performed at least four times, with experimental values expressed as means ± SD. The statistical significance of the differences between the control (absence of CSC) and test (presence of CSC) values was determined by means of a one-way ANOVA. Posteriori comparisons were performed using Tukey’s method. Normality and variance assumptions were verified using the Shapiro-Wilk test and the Brown and Forsythe test, respectively. All of the assumptions were fulfilled. P values were declared significant at ≤ 0.05. The data were analyzed using the SAS version 8.2 statistical package (SAS Institute Inc., Cary, NC, USA).

## Competing interests

The authors declare that they have no competing interests.

## Authors’ contributions

MR and AS conceived the study. AS, KK and HA conducted the experiments. KK, AS, HA, WC, and MR analyzed and interpreted the data. AS, KK and HA drafted the Materials and Methods section. MR completed the manuscript with the help of WC. All of the authors read and approved the final manuscript.
